# Lung Microbiome Dysbiosis in Pulmonary Fibrosis Induced by Multi-Walled Carbon Nanotubes and Bleomycin in Rats

**DOI:** 10.3390/medicina62040688

**Published:** 2026-04-03

**Authors:** Wan-Seob Cho, Muneeswaran Thillaichidambaram, Soyeon Jeon, Gyu-Ri Kim, Sin-Uk Lee, Seung-Ho Lee, Yoon-Ji Kim, Eun-Soo Lee, Youngki Kim, Dongmug Kang, Se-Yeong Kim

**Affiliations:** 1 Lab of Toxicology, Department of Health Sciences, The Graduate School of Dong-A University, Busan 49315, Republic of Korea; wcho@dau.ac.kr (W.-S.C.); tmuneeswaran@hotmail.com (M.T.); wjsthdus0418@naver.com (S.J.); rbfl6692@naver.com (G.-R.K.); dodokook@naver.com (S.-U.L.); 2 Department of Occupational and Environmental Medicine, Pusan National University Yangsan Hospital, Yangsan 50612, Republic of Korea; seung_ho_lee@naver.com (S.-H.L.); es3003@naver.com (E.-S.L.); mungis@pusan.ac.kr (Y.K.); kangdm@pusan.ac.kr (D.K.); 3 Department of Preventive, and Occupational & Environmental Medicine, School of Medicine, Pusan National University, Yangsan 50612, Republic of Korea; harrypotter79@pusan.ac.kr; 4 Research Institute for Convergence of Biomedical Science and Technology, Pusan National University Yangsan Hospital, Yangsan 50612, Republic of Korea

**Keywords:** pulmonary fibrosis, lung microbiome, dysbiosis, multi-walled carbon nanotubes (MWCNTs), bleomycin, inflammation

## Abstract

*Background and objectives*: Occupational and environmental inhalation exposures, including high-aspect-ratio carbon nanotubes, can trigger pulmonary fibrosis (PF). The relationship between exposure-specific fibrogenic pathways (granulomatous inflammation versus diffuse epithelial injury) and lung microbiome dysbiosis remains incompletely understood. We therefore compared lung microbiome alterations in rat PF models induced by multi-walled carbon nanotubes (MWCNTs) and bleomycin. *Materials and Methods*: Female Wistar rats received a single intratracheal instillation of vehicle, MWCNTs (750 μg/rat), or bleomycin (1 mg/rat). At day 28, fibrosis and inflammation were evaluated by histopathology and bronchoalveolar lavage fluid (BALF) profiling. Lung microbial communities were characterized by 16S rRNA gene sequencing (V3–V4). Seventeen lung samples passed stringent quality control and were analyzed (control *n* = 5; bleomycin *n* = 7; MWCNT *n* = 5). *Results*: Both agents induced PF with increased profibrotic signaling, but with distinct pathological signatures: MWCNTs produced localized granulomatous lesions and a robust neutrophilic response (25% of BALF cells), whereas bleomycin caused diffuse interstitial remodeling. Bleomycin increased microbial richness (alpha diversity; *p* < 0.05) and significantly shifted community structure (beta diversity; *p* < 0.05), while MWCNT exposure showed comparatively limited changes in global diversity. The relative abundance of *Pseudogracilibacillus* (including *P. marinus*) was higher in the bleomycin group than in controls, whereas *Facklamia tabacinasalis* and *Corynebacterium maris* were more abundant in the MWCNT group. Across samples, Proteobacteria abundance was inversely correlated with BALF TGF-β, MCP-1, and neutrophil proportion. At the species level, *Pseudogracilibacillus marinus* was positively correlated with BALF TGF-β, while *Facklamia tabacinasalis* and *Corynebacterium maris* were positively correlated with MCP-1, CINC-3, and neutrophil proportion (Spearman; *p* < 0.05). *Conclusions*: Mechanistically distinct fibrogenic exposures generate exposure-linked lung microbiome signatures that track with host inflammatory and profibrotic responses. These signatures may support biomarker development for environmentally and occupationally relevant PF and motivate longitudinal and functional studies to clarify causality.

## 1. Introduction

Pulmonary fibrosis (PF) is a progressive and often fatal interstitial lung disease characterized by repeated epithelial injury, aberrant tissue repair, and excessive collagen deposition in the extracellular matrix. These pathological changes impair alveolar gas exchange and can ultimately lead to respiratory failure [1,2]. Among the various types of PF, idiopathic pulmonary fibrosis (IPF) is the most prevalent and clinically severe form, with a global prevalence estimated to range between 0.33 and 4.51 per 10,000 individuals [3]. Although the precise etiology of IPF remains unclear, multiple factors have been implicated in its development, including aging, genetic predisposition, epigenetic modifications, and environmental exposures [2].

Emerging evidence indicates that the lung microbiome plays a role in the pathogenesis and progression of PF [1,4]. Studies have reported that patients with IPF exhibit increased bacterial burden and dysbiosis within the lower respiratory tract [1,5,6]. High-throughput sequencing technologies have revealed that even healthy lungs possess a diverse and dynamic microbial community [7,8]. Perturbations in this community structure have been associated with several chronic respiratory diseases, including IPF, chronic obstructive pulmonary disease (COPD), asthma, and cystic fibrosis. Specifically, elevated levels of genera such as *Pseudomonas*, *Haemophilus*, *Staphylococcus*, and *Moraxella* have frequently been reported in these conditions [6,9]. Despite these associations, the causal relationship between lung dysbiosis and fibrogenesis remains uncertain. It is not yet clear whether alterations in the microbiome actively contribute to fibrotic remodeling or if they arise secondarily as a consequence of the disease process [4,9]. Additionally, the influence of external factors, such as inhaled toxicants or nanomaterials, on the lung microbiome has received increasing attention [10]. Some studies suggest that dysbiosis itself may exacerbate immune responses and promote disease progression in PF [4,10].

Multi-walled carbon nanotubes (MWCNTs) are widely used in industrial and biomedical fields due to their exceptional mechanical, electrical, and thermal properties [11,12,13]. However, concerns regarding their biocompatibility and safety have emerged [14]. MWCNTs, particularly those with a high aspect ratio and rigidity, have been shown to exhibit asbestos-like pathogenicity in the lungs [14,15,16]. Experimental studies have demonstrated that MWCNTs induce PF through a combination of persistent oxidative stress, inflammasome activation, epithelial damage, and accumulation of myofibroblasts and extracellular matrix components. These pathological features closely resemble those observed in human IPF [17,18]. Bleomycin, an antineoplastic antibiotic, is another well-characterized agent used to induce PF in animal models [19]. It primarily acts through DNA strand breakage and the generation of reactive oxygen species, resulting in alveolar damage and fibrosis [20]. Despite differing mechanisms, both MWCNTs and bleomycin can induce histopathologically similar forms of lung fibrosis [19,21].

In this study, we investigated the impact of PF induced by MWCNTs and bleomycin on the composition and diversity of the lung microbiome in a rat model. By directly comparing a particle-persistent fibrosis model (MWCNT) and a chemically induced fibrosis model (bleomycin), we aimed to determine whether microbial alterations converge into shared fibrosis-associated signatures or diverge according to injury mechanism. This comparative approach is expected to provide insight into whether specific microbiome changes represent a common feature of fibrotic lung remodeling, regardless of the initiating trigger.

## 2. Materials and Methods

### 2.1. Induction of Pulmonary Fibrosis Models in Rats

To investigate the role of the lung microbiome in PF, we employed two well-established fibrosis inducers: MWCNTs and bleomycin. MWCNTs were obtained from Kumho Petrochemical Co., Ltd. (Daejeon, Republic of Korea), and bleomycin was purchased from Sigma-Aldrich (St. Louis, MO, USA). The dispersion of MWCNTs and physicochemical characterization were performed as previously described [15]. Briefly, a stock solution of MWCNTs (15 mg/mL) was prepared in 30% (*v*/*v*) heat-inactivated rat serum collected from six-week-old female Wistar rats. The suspension was sonicated for 80 min using a bath sonicator (40 kHz, 400 W; Saehan Ultrasonic, Seoul, Republic of Korea) to minimize aggregation. Phosphate-buffered saline (PBS, pH 7.4) was then added to obtain a working concentration of 1.5 mg/mL, followed by an additional 10 min sonication. Each rat received an intratracheal instillation of 500 μL (equivalent to 750 μg/rat). Bleomycin was dissolved in PBS (2 mg/mL), and 500 μL was administered via the same route (1 mg/rat). The vehicle group served as a control and received 500 μL of PBS containing 3% rat serum intratracheally. This VEH group served as the common control for both the MWCNT and bleomycin exposure conditions, as the same vehicle background and route of administration were used. Dose selections were based on prior studies demonstrating reproducible fibrosis induction in rodent models [22,23].

### 2.2. Animals and Experimental Design

Six-week-old female Wistar rats were purchased from Central Lab Animal Inc. (Seoul, Republic of Korea) and housed in polycarbonate cages (two animals per cage) under standard laboratory conditions with ad libitum access to food and water. The facility was accredited by the Association for Assessment and Accreditation of Laboratory Animal Care (AAALAC) International (Unit No. 001525). All experimental procedures were approved by the Pusan National University Institutional Animal Care and Use Committee (PNU-2021-0076 on 12 November 2021). After 7 days of acclimatization, 24 rats were randomly assigned to three experimental groups (*n* = 8 per group) to account for possible mortality following exposure to fibrogenic agents. The three groups were as follows: control (PBS + 3% rat serum), bleomycin (1 mg/rat), MWCNT (750 μg/rat).

Intratracheal instillation was performed under isoflurane anesthesia using a rodent anesthesia system (VetEquip, Pleasanton, CA, USA). A 16-gauge catheter was inserted into the trachea, and 500 μL of the test solution was delivered via a 1 mL syringe, following protocols previously described [24]. Twenty-eight days after exposure, all animals were euthanized under deep anesthesia. Lung tissues and bronchoalveolar lavage fluid (BALF) were collected from surviving animals (*n* = 7 in control, *n* = 8 in bleomycin, and *n* = 6 in MWCNT). Lung tissues were processed for microbiome and histopathological analysis, while the BALF was used for biochemical and cytokine assessments.

### 2.3. Biochemical Analysis and Measurement of Pro-Inflammatory Cytokines in BALF

BALF analysis was conducted to characterize PF and changes in inflammatory markers. Lactate dehydrogenase (LDH) and total protein levels in the BALF were measured to evaluate cytotoxicity and vascular permeability, respectively. In addition, the concentrations of pro-inflammatory cytokines in BALF were evaluated using enzyme-linked immunosorbent assay (ELISA) kits (Thermo Fisher Scientific, Waltham, MA, USA): interleukin (IL)-1β, IL-2, IL-4, IL-6, IL-10, cytokine-induced neutrophil chemoattractant-3 (CINC-3), eotaxin, granulocyte–macrophage colony-stimulating factor (GM-CSF), interferon-γ (IFN-γ), monocyte chemoattractant protein-1 (MCP-1), tumor necrosis factor-α (TNF-α), and transforming growth factor-β (TGF-β).

### 2.4. Histological Assessment of Lung Fibrosis

Non-lavaged lung tissues were fixed by inflation with 10% neutral buffered formalin and processed using standard histological techniques at the Neuroscience Translational Research Solution Center (Busan, Republic of Korea). Hematoxylin and eosin (Sigma-Aldrich, St. Louis, MO, USA) (H&E) staining was performed to evaluate overall lung morphology and inflammatory cell infiltration. To visualize collagen deposition and evaluate the histopathological features of pulmonary fibrosis, sections were stained with Picrosirius Red (Sigma-Aldrich, St. Louis, MO, USA) according to the manufacturer’s instructions.

### 2.5. DNA Extraction, 16S rRNA Gene Sequencing, and Sequence Processing

Total DNA was extracted from lung tissue using the DNeasy PowerSoil Kit (Qiagen, Hilden, Germany) according to the manufacturer’s instructions and quantified using the Quant-iT PicoGreen assay (Thermo Fisher Scientific, Waltham, MA, USA). The V3–V4 region of the 16S rRNA gene was amplified following Illumina’s 16S Metagenomic Sequencing Library Preparation protocol. Amplicons were quantified using the TapeStation D1000 system (Agilent Technologies, Santa Clara, CA, USA) and qPCR-based library quantification kits (KAPA Biosystems, Wilmington, MA, USA), and paired-end sequencing (2 × 300 bp) was performed on an Illumina MiSeq platform (Macrogen, Seoul, Republic of Korea). Raw reads were trimmed with Cutadapt v3.2 [25] and denoised with DADA2 v1.18.0 in R software version 4.0.3 (R Foundation for Statistical Computing, Vienna, Austria) to infer amplicon sequence variants (ASVs), including chimera removal. Samples were rarefied to the minimum sequencing depth for diversity analyses using QIIME v1.9 [26]. Taxonomy was assigned using BLAST+ v2.9.0 (National Center for Biotechnology Information, Bethesda, MD, USA) [27] against the NCBI 16S Microbial database using predefined identity and coverage criteria, and a phylogenetic tree was generated using MAFFT v7.475 [28] and FastTreeMP v2.1.10 [29] for downstream analyses.

### 2.6. Bioinformatic and Statistical Analysis

Downstream analyses were conducted using the MicrobiomeAnalyst 2.0 (McGill University, Montreal, QC, Canada) web platform [30]. Low-abundance features (present in <20% of samples with <4 counts) and low-variance features (lowest 10% interquartile range) were filtered, and the remaining features were normalized using total sum scaling (TSS). Of 21 lung tissue samples submitted for sequencing, 17 passed quality control and were included in the final analysis (control *n* = 5; bleomycin *n* = 7; MWCNT *n* = 5). For diversity analyses, ASV tables were rarefied to the minimum sequencing depth using QIIME v1.9. Alpha diversity (Chao1, Shannon, and Simpson indices) was compared across groups using the Kruskal–Wallis test, followed by post hoc pairwise Mann–Whitney U tests with Holm–Bonferroni correction where appropriate. Beta diversity was assessed by principal coordinate analysis (PCoA) based on Bray–Curtis and Jaccard distance matrices, and group differences were evaluated using PERMANOVA. Differential abundance for pairwise group comparisons was assessed using the Wilcoxon rank-sum test, and hierarchical taxonomic patterns were visualized using heat trees. Biochemical and cytokine data were analyzed using GraphPad Prism v9.0 (GraphPad Software, La Jolla, CA, USA) using the Kruskal–Wallis test for multi-group comparisons and the Mann–Whitney U test for pairwise comparisons. Associations between bacterial relative abundance (phylum, genus, and species levels) and PF-related endpoints, including BALF cytokines and differential cell profiles, were evaluated using Spearman’s rank correlation. Statistical significance was set at *p* < 0.05.

## 3. Results

### 3.1. Physicochemical Characterization of MWCNT

**Figure 1:**
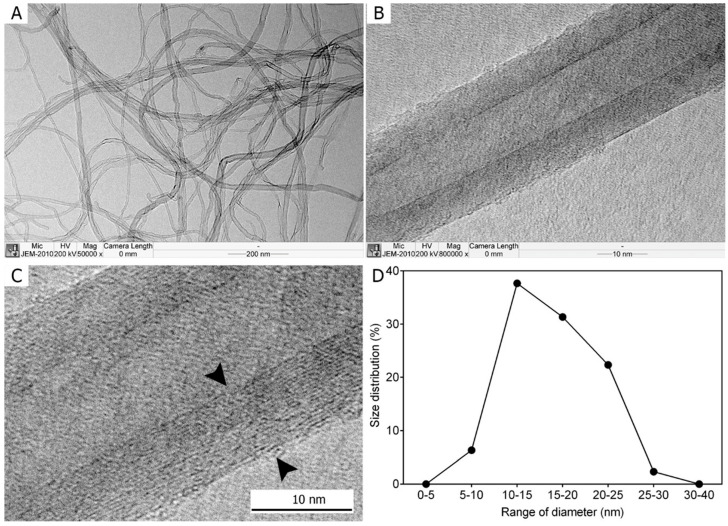
Morphology of the multi-walled carbon nanotubes (MWCNTs) used in the study evaluated using transmission electron microscopy. (**A**) Low magnification (×50,000) shows that MWCNTs are curly but definable for each fiber. (**B**) High-magnification imaging (×800,000) shows that MWCNTs exhibit a tubular form. (**C**) The high-magnification image demonstrates that fibers comprise >20 layers. (**D**) The diameter distribution of fibers indicates that the fiber diameter ranges from 5–30 nm, and the mean diameter is 16.37 ± 0.25 nm.

Transmission electron microscopy (TEM) revealed that the MWCNTs used in this study exhibited a well-defined tubular morphology. At low magnification (×50,000), individual fibers were curly but distinguishable, while high-magnification imaging (×800,000) confirmed their multi-layered tubular structure, comprising more than 20 layers. The fiber diameters ranged from 5 to 30 nm, with a mean diameter of 16.37 ± 0.25 nm (Figure 1). The median fiber diameter was 15.74 nm. The length of the MWCNTs could not be precisely determined but was ~3.69 μm. The tested MWCNT exhibited a high-aspect-ratio, fiber-like morphology, which distinguishes it morphologically from many conventional non-fibrous industrial particulates.

### 3.2. Changes in Inflammatory Markers Induced by MWCNT and Bleomycin Evaluated by BALF Analysis

**Figure 2:**
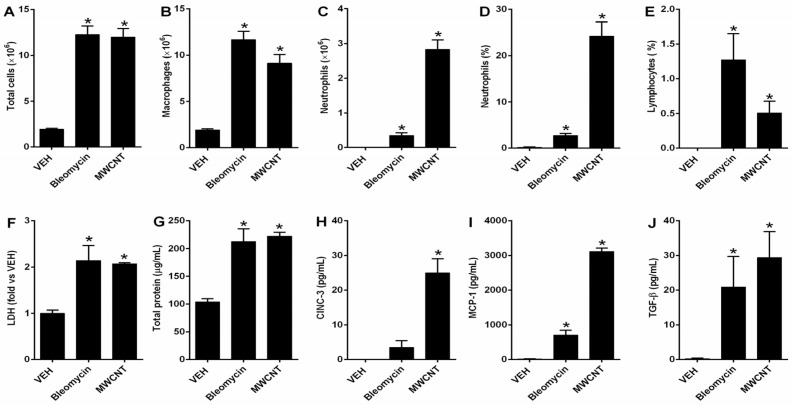
Changes in inflammatory markers induced by multi-walled carbon nanotubes (MWCNT) and bleomycin evaluated by bronchoalveolar lavage fluid (BALF) analysis. (**A**) Number of total cells; (**B**) number of macrophages; (**C**) number of neutrophils; (**D**) percentage of neutrophils among total cells; (**E**) percentage of lymphocytes among total cells; (**F**) lactate dehydrogenase (LDH) level; (**G**) total protein concentration; (**H**) cytokine-induced neutrophil chemoattractant-3 (CINC-3) concentration in BALF; (**I**) monocyte chemoattractant-1 (MCP-1) concentration in BALF; and (**J**) transforming growth factor-β (TGF-β) concentration in BALF. VEH: shared vehicle control used for comparison with both the MWCNT- and bleomycin-treated groups. Statistical significance was determined using the Kruskal–Wallis test followed by post hoc pairwise Mann–Whitney U tests. * *p* < 0.05 vs. VEH.

At 28 days post-instillation, BALF analysis revealed that both MWCNT and bleomycin significantly induced pulmonary inflammation. Comparisons in Figure 2 were made against the shared VEH control group. The total BALF cell counts were increased approximately 6-fold in both treatment groups compared to the control, primarily due to the recruitment of alveolar macrophages (Figure 2A,B). Neutrophil counts and percentages were also significantly elevated in both groups (Figure 2C,D). Notably, neutrophils accounted for ~25% of total cells in the MWCNT group, markedly higher than the ~3% observed in the bleomycin group (Figure 2D), suggesting a more robust neutrophilic response to MWCNT. Changes in lymphocyte proportion were also observed, although these were less prominent than the neutrophil-dominant inflammatory pattern (Figure 2E). LDH and total protein levels—markers of cytotoxicity and vascular permeability—were increased approximately 2-fold in both MWCNT and bleomycin groups compared to the control, indicating comparable lung injury (Figure 2F,G).

Among the 12 cytokines analyzed, only CINC-3, MCP-1, and TGF-β were significantly elevated in both treatment groups (Figure 2H–J). Levels of CINC-3 and MCP-1 were significantly higher in the MWCNT group than in the bleomycin group, aligning with the enhanced neutrophilic infiltration and more severe chronic inflammation observed in the MWCNT-exposed lungs. Additionally, the elevated TGF-β levels in both groups were consistent with the development of PF.

### 3.3. Histological Evaluation of Lung Injury Induced by MWCNT and Bleomycin

**Figure 3:**
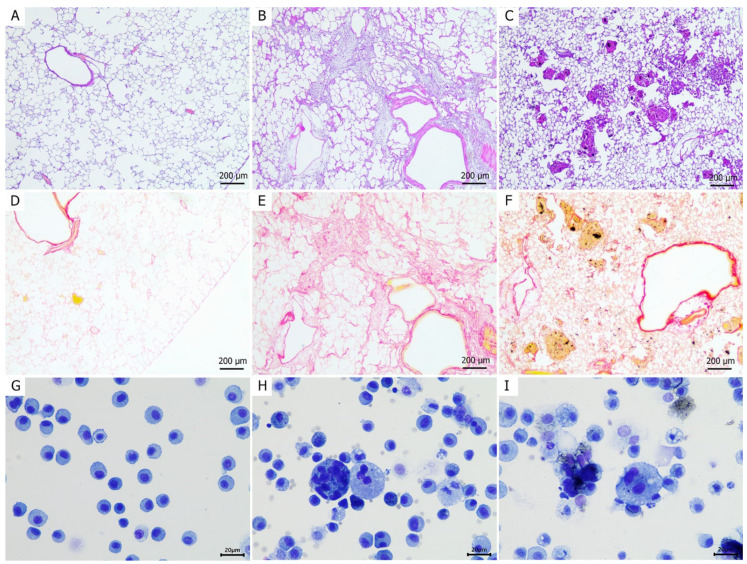
Histological assessment of lung lesions at day 28 post-intratracheal instillation in rats. Histological examination of hematoxylin and eosin-stained lung tissue sections of the (**A**) control, (**B**) bleomycin, and (**C**) multi-walled carbon nanotube (MWCNT)-administered groups. Picrosirius red-stained sections of the (**D**) control and (**E**) bleomycin- and (**F**) MWCNT-administered groups. Photomicrographs of bronchoalveolar lavage fluid (BALF) from the (**G**) control and (**H**) bleomycin- and (**I**) MWCNT-administered groups.

Histological analysis revealed that both MWCNT and bleomycin induced moderate-to-severe lung injury characterized by alveolar and interstitial inflammatory cell infiltration, granulomatous reactions, and fibrotic changes (Figure 3). However, the injury patterns were distinctly different between the two agents.

In the MWCNT-administered group, multiple discrete, round foci of granulomatous inflammation were observed, typically centered around accumulated black MWCNT fibers (Figure 3C). Fibrosis was predominantly localized at the periphery of these granulomas, with collagen fibers appearing to encapsulate the lesions, a characteristic of foreign-body responses (Figure 3F). In contrast, bleomycin administration resulted in a more diffuse fibrotic pattern, with collapsed alveolar architecture and linear collagen deposition in a track-like distribution (Figure 3B,E). Additionally, both treatment groups exhibited perivascular edema and inflammatory infiltrates. Multinucleated giant cells, indicative of chronic inflammation, were also observed in the lungs of both MWCNT- and bleomycin-treated rats (Figure 3H,I). Qualitatively, their histologic context appeared different between groups, with the MWCNT-exposed lungs showing features more consistent with persistent particle-associated chronic inflammation, whereas in the bleomycin group these cells appeared within a broader tissue injury-remodeling background.

### 3.4. Changes in the Composition and Diversity of the Lung Microbiome

Seventeen lung samples passed quality control and were included in the downstream microbiome analyses. Rarefaction curves indicated sufficient sequencing depth (Supplementary Figure S1).

**Figure 4:**
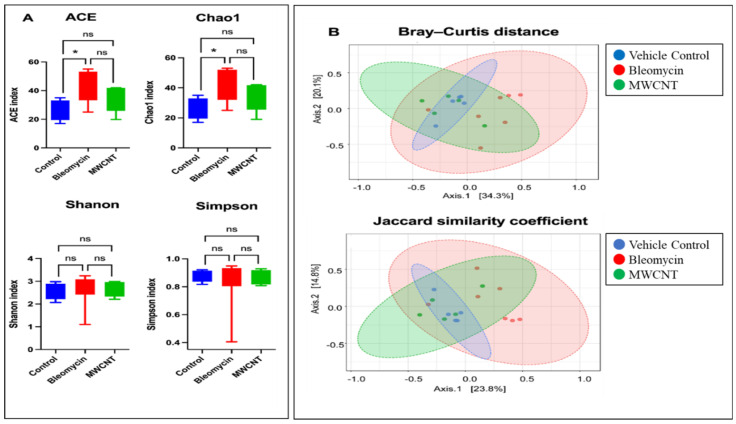
Boxplot of α-diversity indices and principal coordinated analysis plots of β-diversity distance matrices at 4 weeks post-administration with bleomycin and multi-walled carbon nanotubes (MWCNT). (**A**) Abundance-based Coverage Estimator (ACE), Chao1, Shannon index, and Simpson index. (**B**) Principal coordinate analysis with Bray–Curtis distance (F = 2.0604; R^2^ = 0.22741; *p* = 0.024) and Jaccard similarity coefficient (calculated using the differences in less abundant species, irrespective of phylogeny, F = 1.8025; R^2^ = 0.20477; *p* = 0.013). * significantly (*p* < 0.05); ns = No significant.

Alpha diversity analysis showed that the bleomycin-administered group had significantly higher Chao1 and ACE indices compared with the controls, suggesting increased microbial richness. No significant differences in Shannon or Simpson diversity were observed between groups. In contrast, the MWCNT group showed no significant changes in any alpha-diversity indices (Figure 4A). Beta diversity, assessed using Bray–Curtis and Jaccard indices, revealed significant differences in microbial community composition between groups (PERMANOVA, *p* < 0.05), with the most pronounced shift observed in bleomycin-exposed samples compared to controls (Figure 4B).

**Figure 5:**
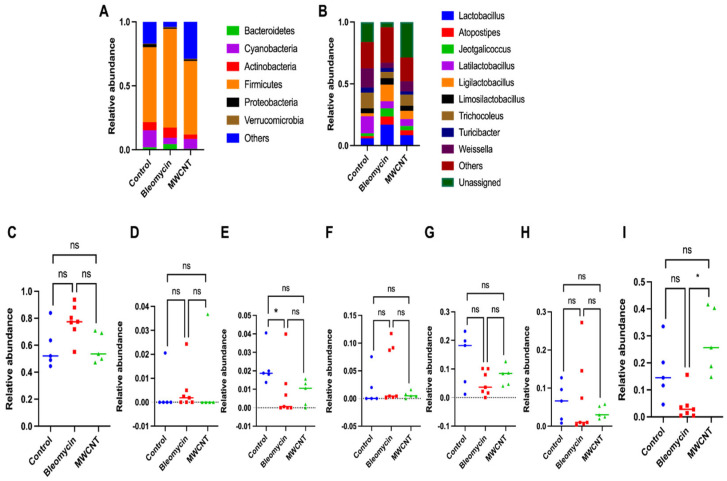
Relative abundance of lung microbiome at 4 weeks post-instillation of bleomycin and multi-walled carbon nanotubes (MWCNT) into the lungs of rats. (**A**) Phylum level. (**B**) Genus level. Only the top ten bacteria at the genus level are presented in the graph, and the others are combined as “Others”. Relative abundance of the microbiome at the phylum level: (**C**) Firmicutes, (**D**) Verrucomicrobia, (**E**) Proteobacteria, (**F**) Bacteroidetes, (**G**) Cyanobacteria, (**H**) Actinobacteria, and (**I**) others. Statistical significance was determined using the Kruskal–Wallis test followed by post hoc pairwise Mann–Whitney U tests. * *p* < 0.05 vs. VEH. ns: not significant.

At the phylum level, six major phyla were identified: Proteobacteria, Firmicutes, Actinobacteria, Bacteroidetes, Cyanobacteria, and Verrucomicrobia. Both treatment groups exhibited a trend toward increased Firmicutes and decreased Proteobacteria and Cyanobacteria compared to the controls; however, these differences were not statistically significant. Notably, only the bleomycin-administered group showed a significant reduction in Proteobacteria abundance compared to the control group (*p* < 0.05) (Figure 5).

**Figure 6:**
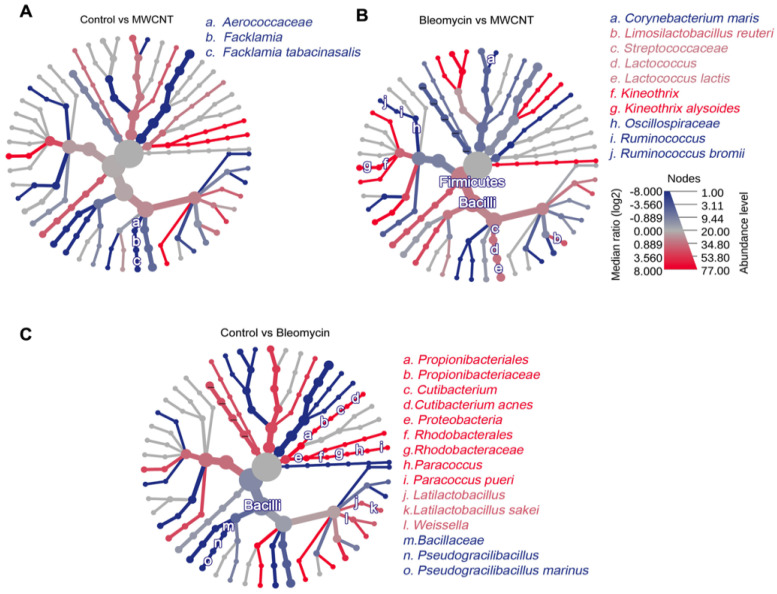
Quantitative comparison of the lung microbiome of rats administered multi-walled carbon nanotubes (MWCNT) or bleomycin. Heat tree analysis demonstrating changes in the microbial composition in the following comparisons: (**A**) Control vs. MWCNT; (**B**) Bleomycin vs. MWCNT; (**C**) Control vs. Bleomycin. Nodes depict the hierarchical structure of taxa, and taxa that are significantly altered are represented by letters. Statistical significance was assessed using the Mann–Whitney U test, with *p* < 0.05 as the cutoff. Red and blue branches indicate higher and lower taxa than the corresponding comparison group, respectively.

Pairwise differential abundance analyses were performed in MicrobiomeAnalyst using the Mann–Whitney U (Wilcoxon rank-sum) test. Compared to the control group, the MWCNT-administered group exhibited a relatively higher abundance of *Facklamia* (genus) and *Facklamia tabacinasalis* (species), whereas the bleomycin-administered group showed an increased abundance of *Pseudogracilibacillus* (genus) and *Pseudogracilibacillus marinus* (species). In contrast, the control group had a relatively higher abundance of *Cutibacterium* (genus), *Cutibacterium acnes* (species), *Latilactobacillus* (genus), and *Latilactobacillus sakei* (species), etc. (Figure 6).

### 3.5. Correlation of Microbial Abundance and Toxicity Endpoints

**Figure 7:**
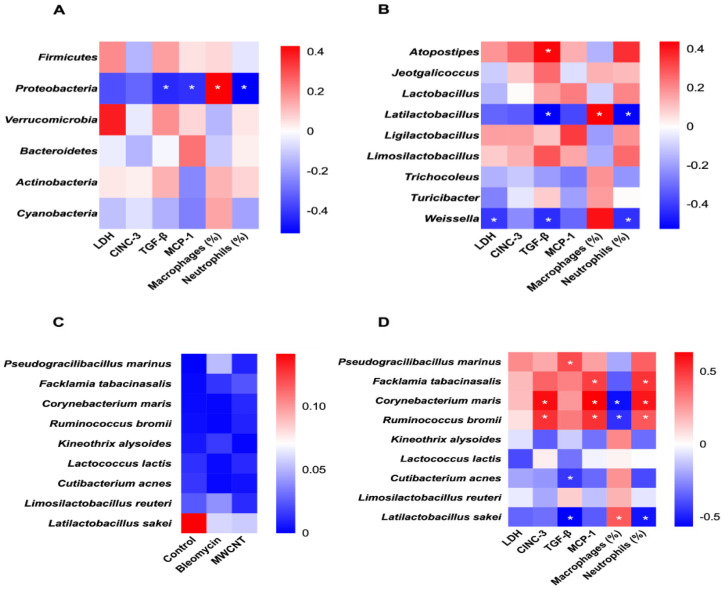
Correlation of microbial abundance and measured toxicity endpoints, such as lactate dehydrogenase (LDH), pro-inflammatory cytokines (cytokine-induced neutrophil chemoattractant-3 [CINC-3], transforming growth factor-β [TGF-β], and monocyte chemoattractant protein-1 [MCP-1]), and cytological data. Correlation of microbiome abundance at the (**A**) Phylum and (**B**) Genus levels with selected toxicological endpoints. (**C**) Heat map of bacterial abundance among the tested groups and (**D**) Correlation of microbiome abundance at the species level. The red color indicates a positive correlation, and the blue color indicates a negative correlation. The degree of correlation between the abundance and the measured toxicological endpoints is expressed by the intensity of the colors (* *p* < 0.05).

Correlations between microbial abundance and markers of pulmonary toxicity were visualized using heatmaps at the phylum, genus, and species levels (Figure 7). At the phylum level, Proteobacteria abundance was significantly negatively correlated with TGF-β, MCP-1, and neutrophils (%), but positively correlated with macrophages (%) (Figure 7A).

Among the top ten genera, *Atopostipes* showed a significant positive correlation with TGF-β, while *Latilactobacillus* and *Weissella* were negatively correlated with TGF-β and neutrophils (%). Additionally, *Latilactobacillus* showed a positive correlation with macrophages (%), and *Weissella* was significantly negatively associated with LDH (Figure 7B).

At the species level, nine taxa identified from previous analyses (Figure 6) were examined. Six of these showed significant correlations (Figure 7D). *Pseudogracilibacillus marinus* was positively correlated with TGF-β, while *Cutibacterium acnes* showed a negative correlation. *Corynebacterium maris* and *Ruminococcus bromii* were positively correlated with CINC-3, MCP-1, and neutrophils (%), but negatively with macrophages (%). *Facklamia tabacinasalis* was positively correlated with MCP-1 and neutrophils (%). *Latilactobacillus sakei* showed a negative correlation with TGF-β and neutrophils (%), alongside a positive correlation with macrophages (%).

## 4. Discussion

This study demonstrates that both MWCNT and bleomycin exposure lead to chronic pulmonary inflammation and fibrosis in rats, but with distinct immune responses and microbial alterations, likely reflecting differences in their pathophysiological mechanisms. BALF and histopathological analysis confirmed that both MWCNT and bleomycin exposure induced marked inflammatory responses and fibrosis. Notably, MWCNT exposure resulted in a significantly higher proportion of neutrophils and increased levels of CINC-3 and MCP-1 compared to bleomycin, indicating a more robust chronic inflammatory response. Although lymphocyte changes were not the dominant feature in BALF cellular profiles, they may still reflect adaptive immune involvement in the evolving inflammatory and fibrotic microenvironment. This aligns with prior studies showing that high-aspect-ratio carbon nanomaterials provoke persistent immune activation and granuloma formation [10,14]. The presence of granulomatous inflammation surrounding retained MWCNT fibers and peripheral fibrosis suggests a foreign-body-type chronic inflammatory mechanism, distinct from the diffuse epithelial injury and track-like fibrosis observed with bleomycin [18,19]. This difference may also reflect the distinctive fiber-like morphology of MWCNTs compared with many conventional industrial particulates. In this context, the multinucleated giant cells observed at day 28 may also reflect different chronic inflammatory settings between the two models. In the MWCNT group, they are more compatible with a persistent particulate/foreign body-type response, whereas in the bleomycin group they may be interpreted within a tissue injury–repair process rather than a clearly particle-persistent reaction. While bleomycin induces fibrosis primarily via oxidative stress, DNA damage, and epithelial apoptosis [19,20], MWCNT appears to trigger fibrotic cascade involving frustrated phagocytosis, inflammasome activation, and long-term retention in the lung tissue [15,16,17,31]. These distinct injury profiles highlight the importance of considering material-specific mechanisms in pulmonary toxicology.

Microbial sequencing revealed that bleomycin exposure significantly increased α-diversity indices (Chao1, ACE), suggesting greater microbial richness, possibly due to compromised epithelial integrity and expanded colonization by microbial niches. This observation aligns with results from previous bleomycin-induced PF rat models [32], suggesting that it is due to an increased bacterial burden. However, it does not align with findings in human studies that reported reduced α-diversity in PF [1,5,6,8], indicating that this response may be model-specific or time-dependent [6]. In contrast, MWCNT exposure did not significantly alter either α- or β-diversity, indicating a relatively preserved microbial homeostasis despite the presence of inflammation. This may be attributed to the localized and compartmentalized nature of the granulomatous lesions induced by MWCNTs [10]. Beta diversity analyses further supported these distinctions, with significant shifts in microbial community structure observed in the bleomycin-exposed lungs but not in the MWCNT-treated ones. This observation aligns with prior findings that epithelial injury and altered pulmonary microenvironments can disturb microbial equilibrium and promote dysbiosis [33]. The clinical implications of increased or decreased microbial diversity in IPF remain controversial, with some studies associating decreased diversity with worse prognosis [5]. In contrast, others report increased diversity in rapidly progressive cases [4,34]. This highlights that microbial diversity metrics must be interpreted in the context of the host’s immune status and the functional role of specific taxa.

Taken together, these findings should be interpreted in light of important differences between experimental fibrosis models and human interstitial fibrotic lung disease, particularly idiopathic pulmonary fibrosis (IPF). Human IPF is a chronic and heterogeneous disorder shaped by aging, repeated epithelial injury, comorbidities, and prolonged host–microbiome interactions [2,4,8]. Human studies have shown that IPF is associated with altered lung microbial composition, impaired microbial diversity, and increased bacterial burden, which have been linked to disease progression and local profibrotic inflammatory responses [1,5,34]. These findings support the concept that host–microbiome interactions may contribute to fibrotic remodeling rather than simply reflect end-stage structural damage [1,4,8]. In contrast, the present rat models reflect controlled fibrosis induction after specific experimental exposures and therefore may not fully reproduce the chronic complexity of human disease. Such differences may partly explain why changes in lung dysbiosis, inflammatory cell profiles, and fibrosis-related biomarkers observed in animal models do not always align with those reported in human studies. Nevertheless, comparative animal models remain useful for identifying exposure-linked host–microbiome interactions and mechanistic pathways. From a translational perspective, microbiome-associated signatures may ultimately contribute to fibrotic lung disease endotyping, biomarker discovery, and the development of exposure-informed therapeutic strategies, although these implications require validation in human longitudinal studies [4,8,34].

Correlation analyses revealed that certain microbial taxa were closely associated with fibrotic and inflammatory markers. At the phylum level, Proteobacteria were negatively correlated with TGF-β, MCP-1, and neutrophils, but positively with macrophages. This finding stands in contrast to the prevailing view in human IPF studies, where an enrichment of Proteobacteria is often associated with pathobionts and disease progression [1,9]. However, our results align with several animal studies suggesting that Proteobacteria constitute a major portion of the healthy lung microbiota in rodents [35,36,37]. Consequently, the negative correlation observed between Proteobacteria and fibrotic markers like TGF-β may reflect a shift away from a healthy microbial baseline as the disease progresses, rather than a direct anti-fibrotic effect of the phylum as a whole.

At the genus level, *Weissella* and *Latilactobacillus* exhibited inverse correlations with TGF-β and neutrophil levels, indicating potential anti-inflammatory effects. Previous studies have shown that strains such as *Weissella cibaria* possess immunomodulatory properties through the regulation of the nuclear factor kappa-light-chain-enhancer of activated B cells (NF-κB) and mitogen-activated protein kinase (MAPK) signaling pathways [38,39], while *Latilactobacillus curvatus* and *L. sakei* demonstrate probiotic traits including antioxidant activity and immune homeostasis support [40,41]. Conversely, *Atopostipes* showed a positive correlation with TGF-β and was enriched in both treatment groups. While *Atopostipes* is traditionally characterized as an environmental genus frequently isolated from livestock manure, soil, and dust [42], it may serve as an opportunistic colonizer that thrives when the lung’s healthy microbial baseline and clearance mechanisms are disrupted.

At the species level, *Facklamia tabacinasalis* and *Corynebacterium maris* were positively associated with pro-fibrotic markers and were more abundant in MWCNT-exposed lungs. Although their direct roles in fibrogenesis remain unclear, these taxa may reflect opportunistic colonization or ecological shifts in chronically inflamed/remodeling lungs; this interpretation is consistent with limited taxonomic and clinical reports [43]. Ruminococcus bromii, a well-known gut-associated anaerobe involved in fermentation of resistant starch, was detected in fibrotic lung tissue, suggesting potential microbial translocation or systemic effects mediated via the gut–lung axis [32,44]. However, its role in pulmonary fibrogenesis remains unclear.

In contrast, *Cutibacterium acnes* and *Latilactobacillus sakei* were more abundant in the control group. They showed negative correlations with key fibrotic markers such as TGF-β and neutrophils, suggesting a potential homeostatic association in the lung environment. While *C. acnes* is traditionally recognized for its association with skin disorders like acne [45] and certain systemic conditions like sarcoidosis and opportunistic infections in immunocompromised hosts [46,47], its role in human health is increasingly viewed as more complex. Emerging evidence indicates that *C. acnes* is not merely a pathogen but also functions as a homeostatic agent that contributes to immune modulation and the maintenance of epithelial barrier integrity [48]. This dualistic nature suggests that the presence of *C. acnes* in the lung may vary in its impact, ranging from opportunistic pathogenicity to protective commensalism depending on the host microenvironment. Similarly, *L. sakei*, a well-characterized lactic acid bacterium, has demonstrated potent anti-inflammatory, immunomodulatory, and antiviral properties in both gastrointestinal and respiratory contexts [49,50]. In the present study, its inverse correlation with fibrotic markers aligns with previous reports highlighting its probiotic potential, further suggesting the feasibility of its therapeutic application in chronic lung diseases characterized by microbial dysbiosis and persistent inflammation.

A major strength of this study lies in its integrated multi-level approach, combining histopathological, cytological, and immunological evaluations with comprehensive 16S rRNA-based lung microbiome profiling. By directly comparing two mechanistically distinct fibrogenic agents—MWCNT and bleomycin—in a controlled animal model, the study offers unique insights into agent-specific immune activation and microbiota perturbation. Importantly, the correlation analysis between key fibrotic/inflammatory markers (TGF-β, MCP-1, neutrophils, LDH) and microbial taxa across multiple taxonomic levels (phylum, genus, species) represents a notable advancement, as few studies have attempted to link host immunopathology with specific microbial signatures in lung fibrosis. These findings provide a novel perspective on potential microbiome-derived biomarkers or modulators of PF.

However, several limitations should be acknowledged. First, the modest sample size (17 lung samples after sequencing QC) may have limited power to detect subtle shifts, particularly for low-abundance taxa, and increases the risk of false-positive findings in feature-level analyses. Second, this study profiled lung tissue microbiota using 16S rRNA amplicon sequencing, which provides limited functional resolution and may not reliably discriminate closely related taxa at the species level; thus, taxonomic signals should be interpreted as hypothesis-generating and validated with higher-resolution approaches (e.g., shotgun metagenomics or targeted qPCR). Third, lung microbiome profiling represents a low-biomass setting; therefore, reagent or environmental contamination and batch effects are potential confounders, and future work should incorporate and report multiple negative controls and contamination-aware filtering to strengthen inference. Fourth, the study employed a single terminal time point (28 days post-instillation), precluding temporal assessment of whether microbiome changes precede, follow, or co-evolve with fibrosis progression. Finally, intratracheal instillation is a controlled method to induce injury but may not fully recapitulate real-world inhalation exposures, which can differ in deposition patterns and kinetics. Longitudinal sampling, absolute bacterial load quantification, and functional experiments (e.g., microbial depletion/repletion or gnotobiotic validation) will be important to clarify causality and translational relevance.

## 5. Conclusions

In summary, this study shows that MWCNT and bleomycin exposure are associated with distinct lung microbiome patterns in a rat model of pulmonary fibrosis, and that these patterns correlate with fibrosis-related and inflammatory host responses. At the genus and species levels, we observed enrichment of taxa consistent with opportunistic colonization and reduction of taxa potentially linked to microbial homeostasis, supporting a context-dependent relationship between dysbiosis and fibrotic lung remodeling. In addition, the detection of gut-associated taxa in fibrotic lung tissue raises the possibility of gut–lung axis involvement in host responses to inhaled toxicants. Taken together, these findings provide a translationally relevant framework for future mechanistic studies and suggest that lung microbiome features may have value as candidate biomarkers or modulatory targets in pulmonary fibrosis, pending longitudinal and functional validation.

## Data Availability

The original contributions presented in the study are included in the article/Supplementary Material; further inquiries can be directed to the corresponding author.

## Supplementary Materials

The following supporting information can be downloaded at: https://www.mdpi.com/article/10.3390/medicina62040688/s1. Figure S1: Rarefaction curves of 16S rRNA (V3–V4 region) gene sequences from lung samples (subsampled to 2340 reads).

## Abbreviations

PF	Pulmonary Fibrosis
MWCNTs	Multi-Walled Carbon Nanotubes
BALF	Broncho Alveolar Lavage Fluid
IPF	Idiopathic Pulmonary Fibrosis
COPD	Chronic Obstructive Pulmonary Disease
AAALAC	Association for Assessment and Accreditation of Laboratory Animal Care
LDH	Lactate dehydrogenase
H&E	Hematoxylin and eosin
ASVs	Amplicon Sequence Variants
PCoA	Principal Coordinate Analysis
TEM	Transmission Electron Microscopy
ACE	Abundance-based Coverage Estimator
