# Supplementary material for: Lung Microbiome Dysbiosis in Pulmonary Fibrosis Induced by Multi-Walled Carbon Nanotubes and Bleomycin in Rats
Source: Medicina (Kaunas). 2026 Apr 3;62(4):688. doi: 10.3390/medicina62040688 (PMC13117696; doi:10.3390/medicina62040688)
Supplement: Supplementary file 1 [file medicina-62-00688-s001.zip › medicina-4178759-supplementary.pdf]

Supplementary material for

“Rarefaction curves of 16S rRNA (V3–V4 region) gene sequences from lung samples (subsampled to 2340 reads)”

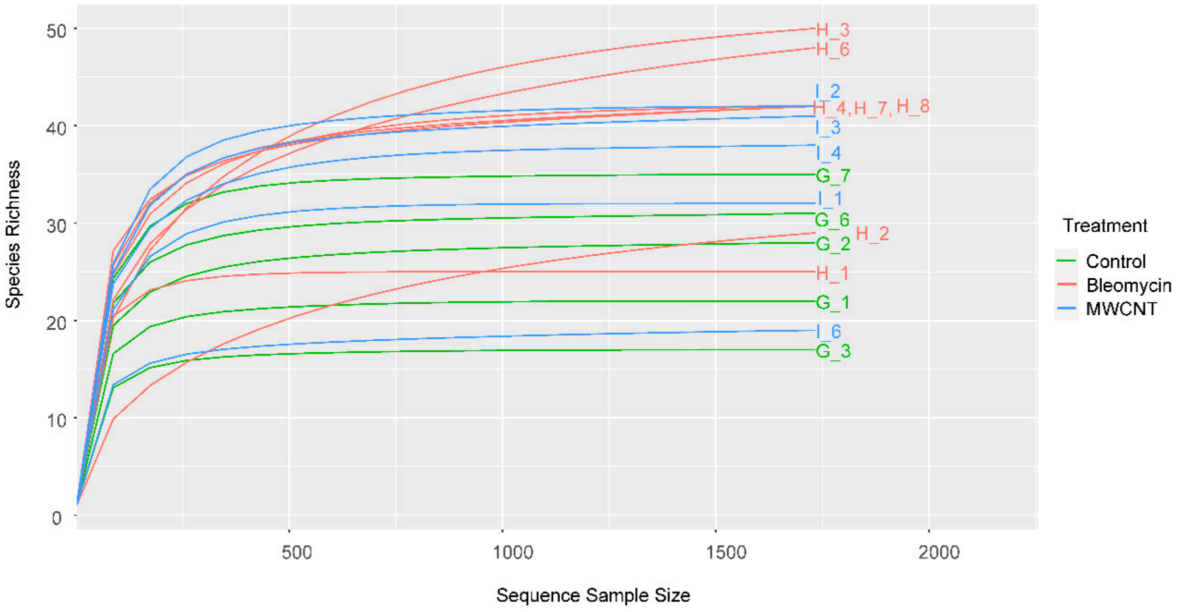

Figure S1. Rarefaction curves of 16S rRNA (V3–V4 region) gene sequences from lung samples (subsampled to 2340 reads). Sample ID G represents the control (n = 5), I represent bleomycin (n = 7), and H represents multi-walled carbon nanotubes (MWCNT) (n = 5).
